# Profile of disability retirement among civil servants at Oswaldo Cruz Foundation, 2012-2016

**DOI:** 10.5327/Z167944352019392

**Published:** 2019-12-01

**Authors:** Marden Samir Santa-Marinha, Liliane Reis Teixeira, Elvira Maria Godinho de Seixas Maciel, Maria de Fatima Ramos Moreira

**Affiliations:** 1 Pos-graduate Program in Public Health and Environment, Sergio Arouca National School of Public Health - Rio de Janeiro (RJ), Brazil. Pos-graduate Program in Public Health and Environment Sergio Arouca National School of Public Health Brazil

**Keywords:** retirement, social security, public sector, insurance, disability

## Abstract

**Background:**

Disability retirement, an outcome of permanent incapacity for work, represents 14.5% of pensions granted by the Brazilian National Social Security Institute. However, there are no data available for civil servants.

**Objective:**

To describe the epidemiological profile of disability retirement among employees of Oswaldo Cruz Foundation (FIOCRUZ) in the period from 2012 to 2016.

**Methods:**

Cross-sectional study in which we analyzed the following variables: age, sex, total working time, years of work at FIOCRUZ, position and reason for retirement as per ICD-10 codes. The significance level was set to p=0.05 and all the data are presented with 95% confidence interval.

**Results:**

The prevalence of disability retirement in the analyzed period was 113/10,000 employees. Employees granted disability retirement benefits worked about 9 years less. Odds for disability were higher for technicians (prevalence ratio-PR=6.83) and technical assistants (PR=7.67). Mental and behavioral disorders were the main reason for disability retirement (38.71%).

**Conclusion:**

Noncommunicable diseases are the main cause of disability retirement. We call the attention to the need to revise the legislation that establishes mandatory retirement after 24 months of sick leave. An interdisciplinary occupational health surveillance approach is necessary to obtain accurate knowledge of the actual situation in workplaces and of the impacts of work processes.

## RESUMO |

**Introdução:**

A aposentadoria por invalidez decorre da incapacidade laboral permanente. Estima-se que essas representem 14,5% do total de aposentadorias do Instituto Nacional do Seguro Social, não havendo dados disponíveis no setor público federal.

**Objetivo:**

Descrever o perfil epidemiológico das aposentadorias por invalidez entre os servidores da Fundação Oswaldo Cruz (FIOCRUZ), no período de 2012 e 2016.

**Métodos:**

Realizou-se um estudo transversal, utilizando-se como variáveis idade, sexo, tempo de trabalho total, tempo de trabalho na Fiocruz, cargo exercido e motivo da aposentadoria segundo capítulo da Classificação Estatística Internacional de Doenças e Problemas Relacionados com a Saúde - 10ª edição (CID-10). Nas análises, um valor de p=0,05 e o intervalo de confiança de 95% (IC95%) foram sempre considerados.

**Resultados:**

A prevalência para aposentadoria por invalidez no período de 2012 a 2016 foi de 113/10.000 servidores. Se aposentar por invalidez representou cerca de 9 anos a menos de trabalho. Trabalhar como técnico (razão das prevalências - RP=6,83) e assistente técnico (RP=7,67) foi ter mais chance de se aposentar por invalidez. Os transtornos mentais e comportamentais foram os que mais motivaram o desfecho, representando 38,71% dos casos.

**Conclusão:**

As doenças crônicas não transmissíveis são as principais causas de aposentadoria por invalidez. Sugere-se reavaliar o dispositivo legal de aposentadoria compulsória aos 24 meses de afastamento. Faz-se necessária uma abordagem interdisciplinar de vigilância em saúde do trabalhador para conhecimento da realidade nos ambientes e o impacto dos processos de trabalho.

## INTRODUCTION

Ours is a globalized work, in which changes in recent years translate, among others, as new information technologies, automation, demands to be continuously connected online, flexibilization of employment relationships, new job descriptions and new ways of organization and division of labor. The resulting social transformations have impact on the lives of all of us^1^.

Work is described as a fundamental means of human expression and transformation into a social being^2^. On these grounds, the human condition is defined based on the central place of work, and well-being on the relationship between work, health and the environment^3^.

However, work can also be a source of disease as a function of the traditional risk factors studied by occupational hygiene and risks inherent to the work process, which are the focus of worker’s health. The result is a historical-social perspective that allows considering the subjectivity of workers and the relevance of psychosocial hazards, leading to a flexibilization of the boundaries between the personal and professional lives^4^^,^^5^.

Sickness absenteeism is a phenomenon that occurs when individuals lose their capacity for work. When permanent, the affected workers are granted disability retirement. Pension is a social benefit established together with the modern industrialized society in the nineteenth century and became a right after many years of work or in the case of disability. Thus it succeeds in keeping retired workers as social subjects with participation in the capital^6^^,^^7^.

Accurate knowledge of the profile of disability retirement benefits is relevant to assess and formulate labor and social protection policies within a context of demographic transition, in which noncommunicable diseases (NCDs) are the main cause of sickness absenteeism. This is a reason for much concern in regard to the sustainability of social security systems^8^^,^^9^^,^^10^.

According to one hypothesis, NCDs account for most cases of disability retirement among the workers in jobs which do not require a high educational level (technicians and technical assistants). A study of disability retirement benefits, therefore, is likely to provide the information necessary to develop intervention strategies relative to the monitoring of workers, compliance with health policies in force, assessment of the health-disease process and the possibilities offered to workers for rehabilitation and thus remain in the labor market.

According to the World Health Organization (WHO), workers with functional aging are those aged 45 or older who in addition to suffering the effects of chronological aging also exhibit biological and psychological impairments which might interfere with their social and professional lives, increase the rate of disease and reduce their total time in the market labor along life^11^.

According to the Brazilian National Social Security Institute, disability retirement accounts for 14.5% of all pensions granted as per the General Social Security Regime. However, there is no information relative to the federal civil servants, who are covered by an ad hoc social security regime, based on the Law no. 8,112/90. This situation makes studies on this subject necessary^1^^,^^12^.

The capacity for work of civil servants is established in medical legal examinations. Workers with permanent disability are suggested to apply for disability retirement, which they may do at any time, but becomes mandatory after 24 months on sick leave^12^^,^^13^^,^^14^.

The aim of the present study was to describe the epidemiological profile of disability retirement benefits granted to Oswaldo Cruz Foundation (FIOCRUZ) employees from 2012 to 2016.

## METHODS

FIOCRUZ is a public institution affiliated to the Ministry of Health which purpose is to carry out actions in health care, education and scientific and technological development to thus consolidate and strengthen the national health system and help improve the quality of life of the Brazilian population^14^.

In 2016 there were 11,852 workers at FIOCRUZ, including civil servants outsourced employees. For the present study, we considered only the Foundation’s effective employees (Law no. 8,112/90), n=5,465, being 3,065 women and 2,400 men. Outsourced employees were excluded due to difficulties to gather information on them.

In the present cross-sectional study we analyzed data obtained from the Pensioner Department and the Medical Legal Examination and Functional Health Assessment Unit, both under FIOCRUZ General Personnel Management Coordination. The time frame considered was the period from January 2012 to December 2016.

Disability retirement was the outcome of interest. As response variables we considered: retirement type (voluntary, disability, mandatory), sex, age, total working years, years working at FIOCRUZ and reason for retirement as per the 10th revision of the International Classification of Diseases (ICD-10). Positions at FIOCRUZ changed over time, therefore, different employees are under different career regimes. We established some equivalences to assimilate all the employees to the five career positions in force: public health researchers, public health technologists, health management analysts, public health technicians and health management technical assistants.

Due to the change in the mandatory retirement age, from 70 to 75, as per the Law no. 152, from 3 December 2015, we did not consider these cases (n=2) in analysis.

Frequencies were analyzed according to sex and position as predictor variables. Assuming that the outcome did not interfere with the selected predictor variables, we calculated prevalence ratios (PR)^15^^,^^16^. Statistical analysis was performed with Statistical Package for the Social Sciences (SPSS) 20.0, for Windows^®^ (SPSS Inc., Chicago, USA). Descriptive analysis included calculation of absolute and relative frequencies, in addition to the prevalence of retirement. The study population did not exhibit normal distribution on the Kolmogorov-Smirnov test, for which reason we calculated medians as measure of central tendency. The significance level was set to p=0.05, and all data are presented with the corresponding 95% confidence interval (95%CI).

As limitations, we call the attention to difficulties to obtain all the required sociodemographic and occupational information, the impossibility of drawing causal inferences due the study design, as well as of generalizing the results as a function of the particular characteristics of the study population. In addition, the number of studies on federal civil servants, health and disability retirement is still small, and the ones available are heterogeneous in objectives, statistical treatment, environment, work process and population, which makes the comparison of results even more difficult. For these reasons, we also calculated means for some variables.

The present study was approved in 25 May 2017 by the institutional research ethics committee, CAAE no. 65658617.4.0000.5240.

## RESULTS

The prevalence of retirement benefits granted along the analyzed period was 816/10,000 employees in general, and 113/10,000 of disability retirement. Table 1 describes the frequency of disability retirement benefits granted per year.

**Table 1.** Distribution of retirement benefits per type and year, FIOCRUZ, 2012-2016 (n=446).

Retirement benefit type		Year					Total (%)
		2012 (%)	2013 (%)	2014 (%)	2015 (%)	2016 (%)	
Voluntary	n	83 (21.6)	90 (23.4)	61 (15.9)	74 (19.3)	76 (19.8)	384 (100.0)
	%	86.5	81.1	83.6	97.4	84.4	100.0
Disability	n	13 (21.0)	21 (33.9)	12 (19.3)	2 (3.2)	14 (22.3)	62 (100.0)
	%	13.5	18.9	16.4	2.6	15.6	100.0
Total	n	96 (21.5)	111 (24.9)	73 (16.4)	76 (17.0)	90 (20.2)	446 (100)
	%	21.5	24.9	16.4	17.0	20.2	100.0

The median age of FIOCRUZ employees was 58 (25th and 75th percentiles 55 and 63). The sample distribution according to age, sex, retirement type and years of work is described in Table 2.

**Table 2.** Distribution of retirement benefits per type, sex, median age and median working time, FIOCRUZ, Brazil, 2012-2016 (n=446).

Retirement benefit type	Sex	N (%)	Median. mean (SD) age at retirement (years)		Median. mean (SD) working time (years)			
					At FIOCRUZ		TOTAL	
Voluntary	Male	144 (37.5)	61	61.4 (5.2)	29	27.4 (9.3)	40	40.1 (5.9)
	Female	240 (62.5)	57.5	58.6 (5.7)	27	25.2 (8.8)	35	34.9 (5.8)
	Total	384	59	59.7 (5.7)	28	26.0 (9.0)	36	36.8 (6.3)
Disability	Male	30 (48.4)	53	52.1 (7.3)	23	19.7 (9.5)	29	30.5 (8.9)
	Female	32 (51.6)	50	51.1 (9.0)	24	21.5 (9.7)	26	2.4 (8.6)
	Total	62	50.5	51.6 (8.2)	24	20.6 (9.6)	27	27.4 (9.2)
Total	Male	173 (39.0)	60	60.0 (6.7)	27	25.3 (9.3)	35	35.5 (7.5)
	Female	272 (61.0)	57	57.7 (6.6)				
	Total	446	58	58.6 (6.7)				

SD: standard deviation

The number of working years was lower, median 9 years, for the employees granted disability retirement compared to those who entered voluntary retirement (p<0.01).

Public health technicians accounted for 53.2% of disability retirement benefits, technical assistants for 21.0%, technologists for 11.3%, management analysts for 9.7% and researchers for 4.8%. The highest frequency of disability retirement benefits corresponded to technicians and technical assistants (p<0.01).

We calculated PR for the variables described in Table 3. Employees in jobs which did not require high educational levels, namely, public health technicians and health management technical assistants, exhibited 6.83 and 7.67 higher odds, respectively, of requiring disability retirement compared to technologists.

**Table 3.** Distribution of retirement benefits per sex and position, FIOCRUZ, Brazil, 2012-2016 (n=5,465).

Variables	Disability retirement		PR	95%CI
	No (%)	Yes (%)		
Sex				
Male	2,370 (98.75)	30 (1.25)	1	
Female	3,033 (98.96)	32 (1.04)	0.84	0.51-1.37
Position				
Researcher	1,047 (99.71)	3 (0.29)	0.76	0.20-2.95
Technologist	1,864 (99.63)	7 (0.37)	1	
Analyst	793 (99.25)	6 (0.75)	2.01	0.68-5.95
Technician	1,259 (97.45)	33 (2.55)	6.83	3.03-15.38
Assistant	440 (97.13)	13 (2.87)	7.67	3.08-19.11

PR: prevalence ratio; 95%CI: 95% confidence interval.

Reasons for disability retirement according to ICD-10 codes are described in Table 4. The most frequent reasons were mental and behavioral disorders (38.71%), followed by musculoskeletal disorders (20.97%), neoplasms and cardiovascular diseases (12.90% each).

**Table 4.** Distribution of disability retirement benefits according to ICD-10 codes, FIOCRUZ, Brazil, 2012-2016 (n=62).

ICD-10 codes	N	%
Mental and behavioral disorders	24	38.71
Diseases of the musculoskeletal system and connective tissue	13	20.97
Neoplasms	8	12.90
Diseases of the circulatory system	8	12.90
Diseases of the genitourinary system	3	4.84
Diseases of the nervous system	2	3.23
Diseases of the eye and adnexa	2	3.23
Endocrine, nutritional and metabolic diseases	1	1.61
Certain infectious and parasitic diseases	1	1.61
Total	62	100.0

Among mental and behavioral disorders, mood disorders accounted for 70.8% of the cases. The prevalence of depressive disorders (ICD-10 F32-33) was 50%, that of bipolar disorder (F31) 16.7%, disorders due to psychoactive substance use 12.5%, psychotic disorders 8.4% and neurotic and somatoform disorders 8.3%.

## DISCUSSION

The number of studies on federal civil servants, health and disability retirement is still small, and the ones available are heterogeneous in objectives, statistical treatment, environment, work process and population, which makes the comparison of results even more difficult.

In recent years, civil servants are stereotypically represented as inefficient and as subjected to a highly hierarchical, rigid and bureaucratic management style. Within such environment there is evidence of mental distress, possibly due to displeasure, depression and illness. However, this population has been scarcely analyzed, which added to difficulties in data collection, represents one further limitation to the discussion of the results of the present study^17^^,^^18^.

The prevalence of retirement benefits we found (11.3/1,000) remarkably differs from that estimated for municipal civil servants in Rio de Janeiro in 2010 (2.80/1,000). In this regard, one should take into consideration differences also in the number of civil servants, jobs and social security legislation^19^.

The fact that mental and behavioral disorders were the main reason for disability retirement corroborates the idea that work environment in civil service might influence the mental health of servants. These conditions were also found to be the main cause for disability retirement in studies conducted in public university in Londrina, Brazil^20^, with retired employees in Kuopio and surrounding rural communities in eastern Finland (16.9%)^21^, Londrina ’s health care workers (45%)^22^, retired employees of Federal University of Minas Gerais, Brazil, (11%)^23^, municipal civil servants in Uberlândia, Brazil (22.6%)^24^ and school principals in Bavaria, Germany (45%)^25^.

Among mental and behavioral disorders, mood disorders demand special attention, since they are a significant reason for retirement, e.g. up to 61% in a study performed with employees of Federal University of Rio Grande do Norte, Brazil, among which depressive disorders accounted for 40% of the cases^26^. These findings confirm the WHO prediction that by 2020 depression will account for the largest burden of disease in terms of disability-adjusted life years (DALY)^27^.

NCDs account for most disability retirement benefits granted, particularly mental and behavioral disorders, musculoskeletal diseases, neoplasms and cardiovascular diseases^24^^,^^28^. These conditions interfere with the productivity of workers and therefore have impact on the social security system. As a function of the intrinsic characteristics of this type of health problems, interventions, and the measurement of their effectiveness, should extend over time, as also health and safety policies to ensure their efficiency^29^.

Differently from the overall situation of women in the labor market in Brazil, most FIOCRUZ employees were female (56.1%). While sick leaves are granted more frequently to female FIOCRUZ employees, the same was not the case of disability retirement, because there was no difference according to sex, as was found in a study performed with employees of Federal University of Minas Gerais, Brazil^10^^,^^23^.

The fact we found a difference of 9 years between the median working time before disability or voluntary retirement might be significant. Since the minimum working time is 30 years for women and 35 years for men, 9 years corresponds to about 1/3 of the total working time.

The median age at the time disability retirement was granted was 50.5. In a study with health care workers at a university hospital in Londrina, Brazil, age at retirement was 48.3±6.26^21^, and the average working time before disability retirement was 17.06±6.26 versus a median of 27 in years in the present study.

Yet, we were not able to establish weather all the working time corresponded to the public sector exclusively, in which area, or also partly in the private sector. When disability retirement follows a medical legal examination, but the workers also meet the criteria for voluntary retirement, they have the right to choose the regime they consider most beneficial to them^12^.

Disability retirement was more frequent among the employees with lower educational level. Similar results were obtained in a study conducted with health care workers in Londrina, Brazil (86.4%) and in Finland (odds ratio 2.44, 95%CI 1.65-3.63). One may assume that workers with higher educational level require disability retirement less often because they have more chances to perform their job in different related settings^22^^,^^30^.

The results obtained might point to a shorter working life and early retirement. These topics should be considered in discussions on functional aging and strategies for occupational risk prevention^11^. The ideal of balance between the workers’ resources and the job demands should inspire effective vocational rehabilitation programs. We could not find any information on implementation or follow-up in this regard. Vocational rehabilitation might serve to assess the potential and performance of employees, thus increasing the odds to help them avoid early and preventable retirement and remain in the labor market.

As a considerable limitation of the present study, we had much difficulty to obtain data relative to a larger number of variables. Studies diverge considerably in methods, populations and variables, which hindered the comparison of results.

## CONCLUSION

NCDs were the main reasons for disability retirement, particularly mental and behavioral disorders. Disability retirement was most frequent among employees in jobs not requiring a high educational level. The total working time was 9 years shorter (median) for disability compared to voluntary retirement. These results indicate that strategies to identify and potentiate the skills and abilities of workers are needed to keep them employed as a means of protection.

Accurate knowledge of the main causes, characteristics factors involved in disability retirement demands an interdisciplinary approach to occupational health surveillance likely to faithfully represent the actual work processes. Only structured surveillance will enable to identify workers’ restrictions, establish the characteristics of the various work environments, perform in-depth analysis of work processes, assess possibilities for reintegration and change the direction of the path leading to disability retirement through novel options.

We also call the attention to the need to revise the legislation on disability retirement, which is currently mandatory after 24 months of sick leave, and to develop means to optimize vocational rehabilitation as an alternative to disability retirement. Space is needed for discussions on health and healthy work processes, as well as databases with information necessary for occupational health surveillance actions for the purpose of achieving work environments likely to help workers preserve their productive life.

## References

[1] Almeida G de FP, Ribeiro MHA, Silva MACN da, Branco RCC, Pinheiro FCM, Nascimento M do DSB (2016). Patologias osteomusculares como causa de aposentadoria por invalidez em servidores públicos do município de São Luís, Maranhão. Rev Bras Med Trab.

[2] Antunes RLC (2011). Adeus ao trabalho? Ensaio sobre as metamorfoses e a centralidade do mundo do trabalho.

[3] Dejours C, Lancman S, Sznelwar L (2004). Christophe Dejours: da psicopatologia à psicodinâmica do trabalho.

[4] Silva JF da, Jardim SR (1997). A danação do trabalho: relações de trabalho e o sofrimento.

[5] Lacaz FA de C (2007). O campo Saúde do Trabalhador: resgatando conhecimentos e práticas sobre as relações trabalho-saúde. Cad Saúde Pública.

[6] Bonamino A, Alves F, Franco C, Cazelli S (2010). Os efeitos das diferentes formas de capital no desempenho escolar: um estudo à luz de Bourdieu e de Coleman. Rev Bras Educ.

[7] Spink MJP, Figueiredo PM, Brasilino J (2011). Psicologia social e pessoalidade.

[8] Camarano AA, Kanso S, Fernandes D (2013). Envelhecimento populacional, perda de capacidade laborativa e políticas públicas.

[9] Ervatti L, Borges GM, Jardim A de P, Instituto Brasileiro de Geografia e Estatística (2015). Mudança demográfica no Brasil no início do século XXI: subsídios para as projeções da população.

[10] Marinha MSS, Teixeira LR, Maciel EMG de S, Moreira M de FR (2018). Perfil epidemiológico do absenteísmo-doença na Fundação Oswaldo Cruz no período de 2012 a 2016. Rev Bras Med Trab.

[11] Organização Mundial da Saúde (2015). Relatório mundial de envelhecimento e saúde - resumo.

[12] Brasil (1990). Dispõe sobre o regime jurídico dos servidores públicos civis da União, das autarquias e das fundações públicas federais. Lei nº 8.112, 1990.

[13] Brasil (1991). Dispõe sobre os Planos de Benefícios da Previdência Social e dá outras providências. Lei nº 8.213, 1991.

[14] Brasil (2014). Manual de Perícia Oficial em Saúde do Servidor Público Federal.

[15] Brasil. Ministério da Saúde (2003). Regimento Interno da Fundação Oswaldo Cruz - Fiocruz. Portaria nº 2.376/GM, 2003.

[16] Szklo M, Nieto FJ (2014). Epidemiology: beyond the basics.

[17] Fonseca R, Carlotto M (2011). Saúde Mental e Afastamento do Trabalho em Servidores do Judiciário do Estado do Rio Grande do Sul. Psicol Pesq UFJF.

[18] Mascarenhas FAN, Barbosa-Branco A (2014). Incapacidade laboral entre trabalhadores do ramo Correios: incidência, duração e despesa previdenciária em 2008. Cad Saúde Pública.

[19] Ferreira NV (2010). Perfil da aposentadoria por invalidez em servidores públicos municipais do Rio de Janeiro de 1997 a 2008.

[20] Moreira AAO, Martins JT, Robazzi ML do CC, Ribeiro RP, Lourenço M do CFH, Lacerda MR (2018). Disability retirement among university public servants: epidemiological profile and causes. Rev Bras Enferm.

[21] Martins JT, Galdino MJQ, Linares PG, Ribeiro RP, Ueno LGS, Broboff MCC (2017). Aposentadoria por invalidez de trabalhadores da área da saúde de um hospital universitário Disability retirement of workers in the health field at a university hospital. Rev Pesqui Cuid Fundam Online.

[22] Karpansalo M, Kauhanen J, Lakka T, Manninen P, Kaplan G, Salonen J (2005). Depression and early retirement: prospective population based study in middle aged men. J Epidemiol Community Health.

[23] Sampaio RF, Silveira AM, Parreira VF, Makino AT, Mateo MM (2003). Análise das aposentadorias por incapacidade permanente entre os trabalhadores da Universidade Federal de Minas Gerais no período de 1966 a 1999. Rev Assoc Med Bras.

[24] Santos AC de Q, Limongi JE, Jorge MLMP, Jorge MT, Pereira BB, Jorge PT (2015). Aposentadorias por invalidez e Doenças Crônicas entre os servidores da Prefeitura Municipal de Uberlândia, Minas Gerais, 1990-2009. Cad Saúde Coletiva.

[25] Weber A, Weltle D, Lederer P (2005). Ill health and early retirement among school principals in Bavaria. Int Arch Occup Environ Health.

[26] Miranda FAN de, Carvalho GRP de, Fernandes RL, Silva MB, Sabino M das GG (2009). Saúde mental, trabalho e aposentadoria: focalizando a alienação mental. Rev Bras Enferm.

[27] World Health Organization (2002). Relatório Mundial da Saúde. Saúde mental: nova concepção, nova esperança.

[28] Vahtera J, Laine S, Virtanen M, Oksanen T, Koskinen A, Pentti J (2010). Employee control over working times and risk of cause-specific disability pension: the Finnish Public Sector Study. Occup Environ Med.

[29] Schramm JM de A, Oliveira AF de, Leite I da C, Valente JG, Gadelha ÂMJ, Portela MC (2004). Transição epidemiológica e o estudo de carga de doença no Brasil. Ciênc Saúde Coletiva.

[30] Polvinen A, Gould R, Lahelma E, Martikainen P (2013). Socioeconomic differences in disability retirement in Finland: The contribution of ill-health, health behaviours and working conditions. Scand J Public Health.

